# Determination of the Ideal Tooth Surface and Pain Threshold to Improve the Efficacy of an Electric Pulp Tester in the Diagnosis of Pulp Sensitivity and Vitality in Premolar and Molar Teeth: A Cross-Sectional Study

**DOI:** 10.7759/cureus.50754

**Published:** 2023-12-18

**Authors:** Taanya Imtiaz, Deepa Gurunathan, Kanamarlapudi Venkata Saikiran

**Affiliations:** 1 Paediatric and Preventive Dentistry, Saveetha Dental College and Hospitals, Saveetha Institute of Medical and Technical Sciences, Saveetha University, Chennai, IND; 2 Paediatric and Preventive Dentistry, Saveetha Dental College and Hospitals, Saveetha Institute of Medical and Technical Sciences, Saveetha University,, Chennai, IND

**Keywords:** innovation, memojis pain scale, pain threshold value, reference sites, pulp sensible test, electric pulp test

## Abstract

Introduction

The electric pulp tester (EPT) is an extensively used diagnostic tool in endodontics. However, several factors, especially the location and thickness of the tooth structures, such as enamel and dentine, can affect the result of an electric pulp test. Further, these factors also alter the pain threshold, which may lead to an inaccurate diagnosis. Hence, it is crucial to ascertain the optimal tooth surface that requires minimal time to elicit a response and pain threshold to enhance the effectiveness of the electric pulp tester for diagnosing the status of the pulp.

Methods

Fifty volunteers (36 males and 14 females) aged 18 to 32 years without any prior experience with the EPT were recruited. The EPT was placed on the seven premolar sites, and molar teeth with an appropriate electrolyte as a conducting medium were tested. The pain threshold values were recorded using the stopwatch, whereas pain assessment was carried out using the Memojis pain scale. An independent sample t-test and descriptive statistics were used to analyze the data statistically.

Results

The buccal occlusal third in males (27.3±8.6 seconds) and the buccal middle third in females (28.5±8.2 seconds) showed lower response times than other sites in premolar teeth. The mesiobuccal cusp showed a lower response time for males (21.3±6.6 seconds) and females (21.5±6.2 seconds) in molar teeth. Of all the various sites tested, the majority of the individuals chose pain scores of 0 (36 in premolars, 84 in molars), two (138 in premolars, 180 in molars), and four (96 in premolars, 42 in molars) in both the premolars and molars.

Conclusion

The ideal sites for placing the EPT in premolars for males and females are the buccal occlusal third and the buccal middle third. At the same time, the mesiobuccal cusp is the ideal site for molars in both males and females, as it is responded to the quickest by the electric current. Most individuals have experienced a score of two (hurts a little bit) for the perceived pain using EPT for both the molars and premolars.

## Introduction

The clinician experiences a significant barrier in accurately diagnosing the pulpal state of posterior teeth affected by caries. The histologic examination, the sole reliable method for assessing the condition of the dental pulp, is not practically viable within the clinical environment [1]. Clinically, the status of the pulp must be determined through clinical examination, intra-oral radiographs, anamnesis, and a pulp sensibility test. These diagnostic approaches are crucial for accurately evaluating and determining suitable treatment options [2].

In dentistry, various diagnostic tests known as pulp sensibility tests are performed to evaluate the sensitivity and vitality of tooth pulp. These tests assist in evaluating pulpal health, identifying any potential problems, and providing the patient with appropriate treatment [3]. The conventional approaches employed for this objective assessment involve cold tests such as refrigerant sprays, ice sticks, CO_2_ snow, and ethyl chloride; warm tests, which include heated gutta-percha and warmed hand instruments; dual wavelength spectrophotometry; laser Doppler flowmetry; estimation of tooth temperature; and pulse oximetry [4]. The electric pulp tester (EPT) is one of the most useful diagnostic tools for the assessment of pulp. This device monitors the nerve's reaction to electrical stimulation, which aids in determining the pulp's health and reveals potential problems such as pulpitis [5].

The patient's perception of the electric stimulus is used to interpret the EPT results. The patient will experience an intense but tolerable sensation, frequently described as tingling or mild discomfort, if the tooth pulp is healthy and vital [6,7]. The nerve may respond at a slower rate if the pulp is injured or inflamed, indicating that irreversible injury or pulp necrosis has occurred [7,8]. In order to reach a precise diagnosis, it is crucial to remember that the EPT is one such tool and that its outcome should be considered in addition to other clinical observations and tests. The data obtained from the EPT may be supplemented with additional diagnostic techniques, such as radiographs and cold or heat sensitivity tests [9].

If used correctly and in the proper circumstances, the EPT is generally considered safe for most patients. Contraindications and other hazards must be taken into account, especially for patients with specific medical disorders or those who could be more susceptible to electrical impulses, such as individuals with cardiac pacemakers and recently erupted teeth with an immature apex [10].

Multiple research studies have indicated that EPT exhibits a notable level of specificity, but its sensitivity varies across various scenarios. In addition to the numerous limitations of the EPT, the need for sufficient information regarding the threshold value also hinders the improvement of its application and clinical interpretation [11]. The EPT threshold value is influenced by various factors, one of which is the thickness of the enamel and dentine [12]. Numerous studies have been conducted to investigate the optimal locations on teeth that exhibit the lowest threshold of response, yielding diverse findings [13,14]. To the best of our knowledge, there is no information in the literature about the level of pain that the individual experienced at various reference sites. Therefore, the current study aimed to identify an optimal tooth surface requiring minimal time to elicit a response and degree of pain using the emojis pain scale participants experienced after placing an EPT tip on both the premolar and molar teeth at various sites.

## Materials and methods

Study setting

The study was conducted in the department of pedodontics at Saveetha Dental College and Hospitals, Chennai, India. Before conducting the study, the institutional ethical review board at Saveetha Dental College and Hospitals, examined and approved the clinical protocol for the study (approval number: IHEC/SDC/FACULTY/23/PEDO/262A).

Seven sites were tested on premolar and molar teeth. The reference sites of the first premolars include the buccal cervical third, buccal middle third, buccal occlusal third, buccal cusp, lingual slope of buccal cusp, buccal slope of lingual cusp, and palatal/lingual surface. The reference sites of the first molars include the mesiobuccal cusp, mesiobuccal cuspal surface, mesiobuccal gingival surface, center of the supporting cusp, distobuccal cuspal surface, distobuccal gingival surface, and center of the guiding cusp.

Participant selection

The sample size estimation was done based on the previous study by Tian SY et al. [14]. A total of 50 individuals, 36 male and 14 female, aged 18 to 32 years, were enrolled in the study based on the following inclusion criteria: teeth free of dental caries, teeth that should not possess any fractured or attrited teeth. Individuals with any history of recent trauma were excluded from the study.

Procedure

All the included participants provided written informed consent before participating in the study after a thorough description and understanding of the study process. After meeting the inclusion criteria, one of the experienced dentists isolated the teeth with cotton rolls and dried them with gauze to prevent salivary contamination. Electric pulp testing was performed using a Waldent electric pulp tester (Waldent Innovations Pvt. Ltd., New Delhi, India) with a range of 0-40 peak of stimulus current reaction numerical value (vital teeth) according to the manufacturer's instructions. After isolation, the electrode tip of the electric pulp tester was coated with toothpaste (Colgate, Colgate Palmolive India Ltd., Mumbai, India), which acts as a conducting medium, and the tip was placed on the surface of the tooth to be examined. The individual was asked to hold the EPT lip clip with their thumb and forefinger to complete the circuit. Participants were instructed to lift their hands upon perceiving a prompt warm, stinging, tingling, or painful sensation. The same investigator recorded the corresponding value on the pulp tester at that precise moment. Finally, a pain assessment was done five minutes after the completion of the procedure using the Memojis pain scale (MPS) at three different sites.

Outcomes and assessment tool

The present study recorded the pulp vitality score on EPT, pain level, and time taken for EPT response for the corresponding tooth surface. On the EPT test, the tooth pulp was considered vital if the patient felt mild pain or a warm, stinging, tingling, or uncomfortable sensation. Further, in those cases where the patient felt pain, the pain was recorded using MPS (score 0 represents no hurt, score 2: hurts a little bit, score 4: hurts a little more, score 6: hurts even more, score 8: hurts a whole lot, score 10: hurts worst). Simultaneously, the time duration between the start of the EPT application and the feeling of pain or tingling sensation was recorded.

This scale comprises six different memoji characters for both males and females, where the males are given an MPS with male memojis and the females with female memojis. The severity of the pain was calibrated on a scale of 0-10. The exact pain score perceived by the individual on the MPS was recorded on a separate paper by the same investigator.

Statistical analysis

The data were entered in Microsoft Excel 2016 (Microsoft Corp., Redmond, WA). The statistical analysis included descriptive statistics, such as the mean and standard deviation. A Mann-Whitney test was performed to compare the mean reaction time between the premolar and molar teeth across the genders. A Kruskal-Wallis test was performed to compare the mean reaction time within the genders. The level of significance was set at 0.05. The data were analyzed using IBM SPSS software version 21.0 for Windows (IBM Corp., Armonk, NY).

## Results

Demographic data

One hundred eighteen posterior teeth (premolars and molars) were included among 50 individuals with a mean age of 26.82 years. There were 666 EPT readings, 336 from premolars, and 330 from molars. All the tested teeth responded positively to the pulp vitality testing using EPT.

Response time

The mean time required to produce the response for the premolars and molars was recorded for both genders using a stopwatch (Tables 1, 2).

**Table 1:** The mean threshold values of the male and female populations at the premolar reference sites in seconds *: significance; SD: standard deviation; #: Mann-Whitney U test; +: Kruskal-Wallis test

Gender	Buccal cervical third (mean±SD)	Buccal middle third (mean±SD)	Buccal occlusal third (mean±SD)	Buccal cusp (mean±SD)	Lingual slope of the buccal cusp (mean±SD)	Buccal slope of the lingual cusp (mean±SD)	Palatal/lingual cusp (mean±SD)	p-value^+^
Male	30.5±8.4	30.8±8.7	27.3±8.6	28.8±9.4	30±9.6	31.1±10.0	37±9.7	0.05*
Female	29.2±8.5	28.5±8.2	30.0±9.6	30.7±9.7	30.2±10.1	31.7±10.6	38.5±11.3	0.05*
p-value^#^	0.44	0.17	0.14	0.32	0.91	0.77	0.47	

**Table 2:** Mean threshold values of the male and female populations at the molar reference sites in seconds MB: mesiobuccal; DB: distobuccal; *: significance; SD: standard deviation; #: Mann-Whitney U test; +: Kruskal-Wallis test

Gender	MB cusp (mean±SD)	MB cuspal surface (mean±SD)	MB gingival surface (mean±SD)	Center of the supporting cusp (mean±SD)	DB cuspal surface (mean±SD)	DB gingival surface (mean±SD)	Center of the guiding cusp (mean±SD)	p-value^+^
Male	21.3±6.6	22.6±5.8	39.1±8.4	28.5±7.2	33.1±7.3	39.5±6.9	43.3±7.7	0.05*
Female	21.5±6.2	22±6.1	37.2±7.9	28.2±7.1	32.7±7.2	39±6.6	42.2±7.2	0.05*
p-value^#^	0.87	0.61	0.24	0.83	0.78	0.71	0.46	

A total of seven sites for both premolars and molars were tested, and the mean response time for all premolar tooth surfaces in males and females ranged from 27.3±8.6 to 37±9.7 seconds and 28.5±8.2 to 38.5±11.3 seconds, respectively. It was higher in females compared to males. In the case of molar teeth, mean response times in males and females ranged from 21.3±6.6 to 43.3±7.7 and 21.5±6.2 to 42.2±7.2 seconds, respectively. Here, it was higher in males compared to females.

The mean response time in premolars was lowest at the buccal occlusal third for males (27.3±8.6 seconds) and the buccal middle third for females (28.5±8.2 seconds). In contrast, the highest mean time was appreciated at the palatal/lingual surface for males (37±9.7 seconds) and females (38.5±11.3 seconds). Similarly, the mesiobuccal cusp of molars has a lesser mean value for both genders (males: 21.3±6.6 seconds; females: 21.5±6.2 seconds). The highest mean time was appreciated at the center of the guiding cusp for both genders (males: 43.3±7.7 seconds; females: 42.2±7.2 seconds).

When these values are compared, the difference is not statistically significant (p>0.05) among the seven different sites tested in both the premolars and molars between the genders. A statistically significant difference was observed across the seven different sites tested in both males and females in both the premolars and molars (p<0.05). 

Pain threshold values for premolars and molars

Table 3 represents the MPS scores perceived by the individuals among the premolars and molars.

**Table 3:** Pain scores perceived by the individuals at various reference points among premolars and molars using the Memojis pain scale

Score	Number of individuals (premolars)	Number of individuals (molars)
0	36	84
2	138	180
4	96	42
6	54	12
8	12	12
10	0	0

The majority of the participants chose a score of two for both the molars (180) and premolars (138), followed by a score of four in the premolars (96) and a score of 0 in the molars (84).

Table 4 represents the MPS scores perceived by the individuals at various reference sites for premolars.

**Table 4:** Pain scores perceived by the individuals at various reference points among premolars

Score	Reference points on premolars						
	Buccal cervical third	Buccal middle third	Buccal occlusal third	Buccal cusp	Lingual slope of the buccal cusp	Buccal slope of the lingual cusp	Palatal/lingual surface
0	6	12	6	-	-	6	6
2	24	24	18	24	-	12	36
4	12	6	6	24	-	6	42
6	6	6	12	18		6	6
8	-	-	6	-	-	-	6
10	-	-	-	-	-	-	-

The majority of the individuals experienced pain scores of two (36) and four (42) on the palatal/lingual surface, and the least painful point of score 0 was appreciated at the buccal middle third (12). Finally, Table 5 represents the MPS scores perceived by the individuals at various reference sites of the molars.

**Table 5:** Pain scores perceived by the individuals at various reference points among molars

Score	Reference points on molars						
	Mesiobuccal cusp	Mesiobuccal cuspal surface	Mesiobuccal gingival surface	Center of supporting cusp	Distobuccal cuspal surface	Distobuccal gingival surface	Center of the guiding cusp
0	24	6	12	12	12	12	6
2	30	12	18	24	36	30	30
4	12	6	6	18	-	-	-
6	12	-	-	-	-	-	-
8	-	6	-	-	-	6	-
10	-	-	-	-	-	-	-

Most individuals experienced a score of two on the distobuccal surface of the molars (36), and the lowest pain score of 0 was observed on the mesiobuccal cusp (24).

## Discussion

The desired characteristics of an ideal pulp test include being easy to use, objective, standardized, reproducible, non-painful, non-injurious, accurate, and cost-effective for evaluating the condition of dental pulp tissue [15]. Pulp testing methods in dentistry may include sensitivity tests such as thermal or EPT to determine the response of the pulp to a stimulus [3]. Electric pulp testing is designed to apply an electric current to stimulate the nearby myelinated A-delta fibers while typically not affecting the unmyelinated C fibers due to their higher threshold. By directing neural transmission, EPT confirms the presence of vital nerve fibers [3, 16]. The electrode of any pulp tester must be appropriately positioned on the tooth's surface at optimal sites to ensure accuracy. Incorrect probe placement may result in false-negative responses in teeth that are actually vital. Electric pulp testing aims to determine each tooth's sensitivity at the lowest threshold for sensory reaction [17]. Electric pulp testing is a valuable tool in diagnosing the vitality of teeth, as it can detect even the slightest response from the nerve fibers. This sensitivity allows for an accurate determination of whether a tooth is vital. Additionally, EPT helps identify the specific threshold at which sensory reactions occur, aiding in further understanding dental sensitivity.

The pulp tester electrode transmits electrical impulses to the pulp primarily through the fluid in the tubules; as the distance between the two entities decreases, the resistance to the flow of electric current decreases correspondingly [18]. These findings support the current study, which found that the mesiobuccal cusp tip closest to the corresponding pulp horn below had a lower electric response [19]. These findings are in accordance with the study conducted by Lin et al. [5]. When determining the best location for the electrode to measure the health of teeth, there are many considerations. The response threshold is attained when numerous nerve terminals are engaged to produce the known summation effect [20]. As the stimulus intensity rises, more sensory nerves become stimulated, gradually raising the sensory response [21]. The activation of sensory nerves is the primary cause of this occurrence. These factors must be considered when deciding where the electrode should be placed to measure tooth vitality effectively.

Based on the current study's findings, the lowest response between premolars was observed either at the buccal occlusal third or the buccal middle third. Filippatos et al. stated that the center of the buccal cusp received the most negligible response among all the tested sites [22]. Tian et al. reported that the buccal cusp of premolars results in a relatively small contact area for the tester tip on the tooth surface, leading to reduced contact stability. Hence, to have more precise and manageable electrical conduction, it is preferable to have a lingual slope on the buccal cusp [14].

The thickness of the enamel and dentine covering the pulp may impact the threshold for response [23]. The relationship between pulp chamber size and enamel thickness, as well as how they affect the mean threshold value, has been studied by several authors [24]. Various studies have yielded inconclusive results about the association between gender and different thresholds [25]. On the other hand, certain studies have provided evidence of gender-related differences in this particular threshold [26]. The current study revealed a statistically significant increase in the average threshold value among female participants for both premolars and molars. In contrast, Tian et al. conducted a study that found that females tended to perceive lower values [14]. One potential hypothesis could be that variations in dentinal thickness, rather than enamel thickness, may account for the observed variances. The presence of thicker dentine may increase the threshold in female participants.

Our findings imply a slight variation between the enamel and dentin covering the pulp chamber in terms of thickness. This is consistent with research that evaluated the lengths of anatomical landmarks in the pulp chambers of human maxillary and mandibular molars and discovered that they were almost identical from the cusp to the pulp chamber ceiling [27]. These findings imply that the size of the pulp chamber may not significantly impact the thickness of the enamel and dentin surrounding the pulp chamber [27]. However, more research is required to examine additional variables that may have an impact on the mean threshold value in connection with pulp chamber size, dentin, and enamel thickness.

Among the different pain scales, MPS was employed in the present study because of its significance in determining pain effectively [28]. Along with this, this is the first gender-based scale developed for males and females, and it is easily understood [28]. The current study has limitations, such as measuring the pulp's health quantitatively and the fact that the EPT readings are based on the pulpal nerves. The present response for different tooth types and sites needs to be studied more to determine how it affects the pulpal size, status, and pattern of innervation. Additionally, future research should consider incorporating a larger sample size to increase the generalizability of the findings. Furthermore, exploring the potential influence of other factors, such as age and previous dental history, on pain perception could provide a more comprehensive understanding of the relationship between tooth types, sites, and pulpal health.

## Conclusions

The study's findings revealed the mean threshold values of EPT for premolars and molars at six different sites in males and females. The buccal occlusal third of premolars in males and the buccal middle third of premolars in females elicited a quicker response with EPT among the various sites tested. The mesiobuccal cusp of molars in both genders has responded quickly to EPT. Irrespective of the site, most individuals have experienced a score of two (hurts a little bit) for the perceived pain using EPT for both the molars and premolars on the Memojis pain scale. Hence, understanding these variations in the EPT values across different regions of the posterior teeth can aid clinicians in accurately assessing the vitality of the teeth.

## References

[1] Igna A, Mircioagă D, Boariu M, Stratul ȘI (2022). A diagnostic insight of dental pulp testing methods in pediatric dentistry. Medicina (Kaunas).

[2] Nagarathna C, Shakuntala BS, Jaiganesh I (2015). Efficiency and reliability of thermal and electrical tests to evaluate pulp status in primary teeth with assessment of anxiety levels in children. J Clin Pediatr Dent.

[3] Chen E, Abbott PV (2009). Dental pulp testing: a review. Int J Dent.

[4] Jafarzadeh H, Rosenberg PA (2009). Pulse oximetry: review of a potential aid in endodontic diagnosis. J Endod.

[5] Lin J, Chandler N, Purton D, Monteith B (2007). Appropriate electrode placement site for electric pulp testing first molar teeth. J Endod.

[6] Turedi I, Ulusoy AT (2022). Evaluation of electric pulp test thresholds and correct probe tip placement site in developing incisors: a clinical study in 1200 teeth. Eur Arch Paediatr Dent.

[7] Richards JF, McClanahan SB, Bowles WR (2015). Electrical pulp testing: sources of error. Northwest Dent.

[8] Chen E, Abbott PV (2011). Evaluation of accuracy, reliability, and repeatability of five dental pulp tests. J Endod.

[9] Patro S, Meto A, Mohanty A (2022). Diagnostic accuracy of pulp vitality tests and pulp sensibility tests for assessing pulpal health in permanent teeth: a systematic review and meta-analysis. Int J Environ Res Public Health.

[10] Sriman N, Prabhakar V, Bhuvaneswaran JS, Subha N (2015). Interference of apex locator, pulp tester and diathermy on pacemaker function. J Conserv Dent.

[11] Owlia F, Mahmoudzade N, Modaresi J, Zarchi MA (2021). Evaluation of the response to electric pulp testing in multiple sclerosis patients without a history of trigeminal neuralgia: a case-control study. BMC Neurol.

[12] Batchelor PA, Sheiham A (2004). Grouping of tooth surfaces by susceptibility to caries: a study in 5-16 year-old children. BMC Oral Health.

[13] Ahmed M, Tegginmani V, Patil D, Ahmed Z, Ahmed N (2020). Evaluation of variability of electric pulp response threshold in molars: an in vivo study. Int J Clin Trials.

[14] Tian SY, Tang L, Zheng CY (2017). Electrode placing sites affect pulp vitality test of human incisors and premolars. Chin J Dent Res.

[15] Caviedes-Bucheli J, Gomez-Sosa JF, Azuero-Holguin MM, Ormeño-Gomez M, Pinto-Pascual V, Munoz HR (2017). Angiogenic mechanisms of human dental pulp and their relationship with substance P expression in response to occlusal trauma. Int Endod J.

[16] Karayilmaz H, Kirzioğlu Z (2011). Comparison of the reliability of laser Doppler flowmetry, pulse oximetry and electric pulp tester in assessing the pulp vitality of human teeth. J Oral Rehabil.

[17] Abd-Elmeguid A, Yu DC (2009). Dental pulp neurophysiology: part 2. Current diagnostic tests to assess pulp vitality. J Can Dent Assoc.

[18] Lampiasi N (2023). The migration and the fate of dental pulp stem cells. Biology (Basel).

[19] Beneng K, Renton T, Yilmaz Z, Yiangou Y, Anand P (2010). Sodium channel Na v 1.7 immunoreactivity in painful human dental pulp and burning mouth syndrome. BMC Neurosci.

[20] Jiménez NT, Carlos Munévar J, González JM, Infante C, Lara SJ (2018). In vitro response of dental pulp stem cells in 3D scaffolds: a regenerative bone material. Heliyon.

[21] Eramo S, Natali A, Pinna R, Milia E (2018). Dental pulp regeneration via cell homing. Int Endod J.

[22] Filippatos CG, Tsatsoulis IN, Floratos S, Kontakiotis EG (2012). The variability of electric pulp response threshold in premolars: a clinical study. J Endod.

[23] Liang C, Liao L, Tian W (2021). Stem cell-based dental pulp regeneration: insights from signaling pathways. Stem Cell Rev Rep.

[24] Xie Z, Shen Z, Zhan P (2021). Functional dental pulp regeneration: basic research and clinical translation. Int J Mol Sci.

[25] Jespersen JJ, Hellstein J, Williamson A, Johnson WT, Qian F (2014). Evaluation of dental pulp sensibility tests in a clinical setting. J Endod.

[26] Chunhacheevachaloke E, Ajcharanukul O (2016). Effects of conducting media and gender on an electric pulp test. Int Endod J.

[27] Deutsch AS, Musikant BL (2004). Morphological measurements of anatomic landmarks in human maxillary and mandibular molar pulp chambers. J Endod.

[28] Saikiran KV, Elicherla SR, Mounika SV, Hemanth Kumar R, Kolavali PS, Nuvvula S (2023). Memojis pain scale: a novel pain assessment tool. Int J Paediatr Dent.

